# Ammonia Induces Autophagy through Dopamine Receptor D3 and MTOR

**DOI:** 10.1371/journal.pone.0153526

**Published:** 2016-04-14

**Authors:** Zhiyuan Li, Xinmiao Ji, Wenchao Wang, Juanjuan Liu, Xiaofei Liang, Hong Wu, Jing Liu, Ulrike S. Eggert, Qingsong Liu, Xin Zhang

**Affiliations:** 1 High Magnetic Field Laboratory, Chinese Academy of Sciences, Hefei, Anhui, P. R. China; 2 University of Science and Technology of China, Hefei, Anhui, P. R. China; 3 Department of Chemistry and Randall Division of Cell and Molecular Biophysics, King’s College London, London, United Kingdom; University of Pittsburgh, UNITED STATES

## Abstract

Hyperammonemia is frequently seen in tumor microenvironments as well as in liver diseases where it can lead to severe brain damage or death. Ammonia induces autophagy, a mechanism that tumor cells may use to protect themselves from external stresses. However, how cells sense ammonia has been unclear. Here we show that culture medium alone containing Glutamine can generate milimolar of ammonia at 37 degrees in the absence of cells. In addition, we reveal that ammonia acts through the G protein-coupled receptor DRD3 (Dopamine receptor D3) to induce autophagy. At the same time, ammonia induces DRD3 degradation, which involves PIK3C3/VPS34-dependent pathways. Ammonia inhibits MTOR (mechanistic target of Rapamycin) activity and localization in cells, which is mediated by DRD3. Therefore, ammonia has dual roles in autophagy: one to induce autophagy through DRD3 and MTOR, the other to increase autophagosomal pH to inhibit autophagic flux. Our study not only adds a new sensing and output pathway for DRD3 that bridges ammonia sensing and autophagy induction, but also provides potential mechanisms for the clinical consequences of hyperammonemia in brain damage, neurodegenerative diseases and tumors.

## Introduction

Ammonia is produced by normal catabolism of proteins and nucleic acids, and at high concentrations can be clinically toxic to the human body, especially to the brain and liver [1–4]. Ammonia is often elevated in human tumor xenografts, as well as in patients with cancer, renal and liver diseases [5–9]. Low millimolar concentrations of ammonia, comparable to the blood ammonia concentration in clinical hyperammonemia patients, tend to reduce cell growth [10]. Recently, ammonia was shown to induce autophagy in cultured cells, and this was proposed to be a mechanism by which tumor cells protect themselves from external stresses, including chemotherapeutics [5,11,12]. However, how cells sense ammonia to induce autophagy still needs to be further explored.

Autophagy is a dynamic process that promotes cellular homeostasis by degradation of protein aggregates and damaged organelles and provision of nutrients [13–15]. Various exogenous cues such as nutritional status, oxygen level or pathogens can all regulate autophagy [16–18]. For example, under starvation, cells can self-digest their less essential components through autophagy to provide nutrients to maintain their vital functions. The most commonly used marker for autophagy is MAP1LC3 (LC3), an ortholog of yeast Atg8 [19], which is also part of the autophagy machinery and is up-regulated upon autophagy induction. Another autophagy specific substrate, SQSTM1/p62, is also frequently used as an autophagy marker because it directly binds to LC3 and is degraded in autolysosomes [20,21]. Increased levels of SQSTM1 are a reliable indicator of suppressed autophagic flux while decreased SQSTM1 levels indicate increased autophagic flux [21,22]. For example, inhibition of MTOR by Rapamycin can increase the lipidated form of LC3, LC3II, and decrease SQSTM1, which is consistent with the suppression role of MTOR in autophagy induction [23,24]. Perturbations of the intra-vesicular pH of autophagy compartments, such as by Bafilomycin A1, Chloroquine or ammonium chloride, inhibit the autophagic flux and cause the increase of both LC3II and SQSTM1.

MTOR is a central regulator of autophagy. Recently, it was shown that GPCRs T1R1 and T1R3 regulate autophagy through MTORC1 in response to amino acids [25]. This discovery linked G-Protein Coupled Receptors (GPCRs) signaling to autophagy activation via MTOR for the first time. The roles of other GPCRs, such as beta adrenergic receptors, in autophagy have also been investigated [26]. As trans-membrane proteins, GPCRs are good candidates to receive extracellular stimuli and correspond with intracellular signal transduction pathways. As the largest membrane receptor family, GPCRs can sense a large variety of ligands, including odorant molecules, peptides, proteins, and even ions and photons [27–31]. Many non-traditional roles of GPCRs have been discovered in recent years [32–34]. For example, Dopamine receptor D3 (DRD3) is not only expressed in brain and neurons, but also in other tissues and cells [35–37] and it plays important roles in endosomal sorting and cytokinesis [35].

While investigating the role of DRD3 in endosomal sorting and cytokinesis, we noticed that the localization of GFP-DRD3-Flag varied between experiments. We became interested in ammonia when we noticed that the behavior of cultured cells expressing Dopamine receptor D3 (DRD3) changed with time after passage. It has been reported before that culture medium that have been incubated with cells for a few days will generate ammonia, which could induce autophagy [5,11]. Although their studies did not observe MTOR activity changes in ammonia-induced autophagy, a recent study using phosphoproteomics shows that phosphorylation of MTOR, S6K as well as EIF4EBP1 are affected by ammonia [12], indicating that there are connections between the MTOR pathway and ammonia. Here, we used a series of time points, concentrations, and cell lines to show that the MTOR pathway is clearly affected by ammonia in a concentration- and time-dependent manner. Recently, Polletta et al reported that SIRT5 controls Glutamine metabolism and the consequent ammonia induced autophagy [38] but the downstream components of ammonia-induced autophagy mechanism is still unclear. We now report that ammonia induce autophagy through DRD3 and MTOR while it also can inhibit autophagic flux by affecting vesicle pH. In addition, DRD3 stability is regulated by autophagy following ammonia exposure.

## Materials and Methods

### Reagents

The anti-GFP (ab290) poly-clonal antibody was from Abcam. The mouse anti-LAMP1 monoclonal antibody (H4A3) was from Developmental Studies Hybridoma Bank. The autophagy antibody sampler kit, the antibodies for SQSTM1/p62 (#5114), pS6K (T389/412), S6K, pEIF4EBP1 (T37/46) (p-4E-BP1), GAPDH, MTOR, EGFR, EIF4EBP1 (4E-BP1), the HRP-linked anti-rabbit and anti-mouse IgG antibody were all from Cell signaling technology. The anti-GFP (sc-9996) antibodies were acquired from Santa Cruz and anti-TUBB (β-Tubulin) and anti-ACTB (β-Actin) antibodies from Beijing TransGen Biotech (Beijing, China). The anti-Flag M2 monoclonal antibody (F3165) was from Sigma. The anti-LC3 (M186-3) monoclonal antibody was from MBL. GlutaMAX supplement, Earle's Balanced Salt Solution (EBSS, 14155–063) and Puromycin Dehydrochloride were from Gibco. The secondary antibodies, anti-fade prolong Gold with DAPI and EGF complexed to AlexaFluro^®^ 555 were from Molecular Probes. The plasmid mCherry-EGFP-LC3 was from Addgene. Pre-stained Protein Ladder (26616) and M-PER buffer were from Thermo Pierce. The cAMP-Glo assay kit was from Promega. Forskolin (FSK), IBMX, Ro 20–1724 were from Sigma. Bafilomycin A1 was from Cayman. Dopamine, Chloroquine, NH_4_Cl and L-Glutamine were from Sigma. VPS34-IN1 was synthesis as previously described [39]. E-64d (E8640-250UG) was from Sigma and used as 10 μg/ml (1 mg/ml stock in ethanol). Pepstatin A (Ultra pure grade, J583-5MG) was from Amresco and used as 68.6μg/ml (6.86 mg/ml in DMSO). The siRNAs were ordered from Genepharm (Shanghai, China). Hiperfect was from Qiagen and Fugene 6 from Promega. Protease inhibitor and Phosphatase inhibitor cocktails were from Roche and the PVDF membrane from Millipore.

### Cell culture and generation of stable cell lines

HeLa, HCT116, CHO-K1 and HEK293T cells were cultured in DMEM supplemented with 10% FBS and 1% penicillin/streptomycin (P/S). HeLa cells stably expressing GFP-DRD3-Flag, GFP-DRD3, DRD3-GFP-Flag, EGFP-LC3-Flag or mCherry-EGFP-LC3 or CHO cells stably expressing GFP-DRD3-Flag were established using retrovirus system. Retroviruses were packaged by transfecting the plasmid inserted the indicated genes with two helper plasmids into 293T cells using Fugene 6 (Promega) and the supernatant containing viruses were harvested after 48 hours. Stable cell lines were generated by infection of HeLa and CHO-K1 cells using MSCV viruses and screened by 1 and 10 μg/ml puromycin and the stable cell lines were maintained in medium containing 1 μg/ml puromycin.

### Immunofluorescence

HEK293T, CHO-K1, HCT116, HeLa or HeLa-GFP-DRD3-FLAG cells were grown on coverslips and treated with the drugs for indicated time points. For cell starvation assay, HeLa-GFP-DRD3-Flag cells were grown on coverslips and washed with warm PBS gently twice and then added new medium, EBSS, or EBSS combined with indicated drugs for 2 hours. Cells were washed once with PBS and fixed by -20°C methanol for 5 minutes or 4% formaldehyde at room temperature for 20 minutes and then blocked by AbDil-Tx (TBS-Tx supplemented with 2% BSA and 0.05% sodium azide) at room temperature for 30 minutes, followed by primary antibodies (Flag, LC3B, LC3, EGFR, LAMP1 (Developmental Studies Hybridoma Bank, H4A3), MTOR) incubation at 4°C overnight. The secondary fluorescently conjugated antibodies were incubated at room temperature for 1 h and washed by TBS-Tx (TBS added with 0.1% Triton x-100) and mounted by anti-fade prolong Gold with DAPI. Images were taken using a Leica DMI4000B fluorescent microscope and the confocal microscope Zeiss LSM710 and analyzed by ZEN 2009 Light Edition and Photoshop. All experiments were repeated at least three times and representative micrographs are shown in the Figures.

### cAMP-Glo assay

The intracellular cAMP level was monitored by the cAMP-Glo assay (Promega) based on the reciprocal relationship between the cAMP concentration and the bioluminescence value. The decreased luminescence reading reflects higher cAMP level in cells. Briefly, 5000 cells (CHO and CHO-GFP-DRD3-Flag cells) were plated in white 384-well plate (Corning, 3570) 24 hours prior to the assay. Cells were washed once with PBS and then were pre-treated with 20 μl compounds of interest in PBS for 25 minutes before treated with 7.5 μl compounds in the presence of 1 mM IBMX, 200 μM Ro 20–1724 and 10 μM forskolin for 15 minutes at room temperature. The subsequent steps were performed as the manufacture’s protocol. The data were acquired with the Multimode Plate Reader (PerkinElmer, EnVision) and analyzed by GraphPad Prism 5 using two-way ANOVA analysis followed by Bonferroni post-test.

### RNAi assay

HeLa or HeLa-GFP-DRD3-Flag cells were plated in 12-well or 24-well plate and 40 or 80 nM siRNAs for *DRD3*, *PIK3C3/VPS34* or negative control were transiently transfected using Hiperfect following manufacture’s protocol. The siRNA sequence for *DRD3* was GUACAGCCAGCAUCCUUAA, for *PIK3C3/VPS34* was ACGGUGAUGAAUCAUCUCCAA. After 72 hours incubation, the cells were either lysed for Western Blotting or fixed to perform immunofluorescence experiments.

### *In vitro* kinase assay

The MTORC1 complex was co-immunoprecipitated with anti-RAPTOR antibody in the lysate of RAPTOR overexpressing HEK293T cells. Purified EIF4EBP1 was used as substrate. The in *vitro* MTORC1 kinase assay was carried out as previously described [40].

### Live cell imaging of HeLa-mCherry-EGFP-LC3 cells

HeLa-mCherry-EGFP-LC3 cells were seeded into the Glass Bottom Culture Dishes (35 mm petri dish, 14 mm Microwell, MatTek Corporation) 24 hours before the experiment. Cells were treated with 5 mM ammonium chloride for 2 hours at 37°C, 5% CO_2_ incubator. Subsequently, cell culture medium was replaced with PBS alone or PBS containing 5 mM ammonium chloride to better visualize the cells. The live cells were then subjected to live cell imaging by the Leica microscope using the GFP and N2.1 channels.

### EGFP-LC3-FLAG cloning

A plasmid containing an N-terminal mCherry-EGFP-LC3 fusion with a pBABE-puro retroviral vector was from Addgene and was previously characterized. Oligonucleotide primer pairs of LC3S (ACTGACCTCGAGCGCCACCATGGTGAGCAAGGGCGAGGAGCTG) and LC3A (ACTGACGCGGCCGCCACTGACAATTTCATCCCGAACGTCTCCTG) were used to subclone EGFP-LC3. A PCR product of 1200 bp was digested with XhoI and NotI and ligated into a mammalian expression retroviral vector pMSCVpuro (Clontech) modified by an insertion of a 3×FLAG sequence at the end of the multiple cloning site (MCS).

### EGF Alexa555 pulse and chase assay

HeLa cells were plated on coverslips and treated with 400 ng/ml EGF Alexa555 for 10 minutes to allow EGF combination to EGFR at 37°C. After 10 minutes, cells combined with EGF Alexa555 were washed with PBS and chased in medium without EGF Alexa555 at 37°C for 45 minutes before fixed images were taken.

### Western blotting

Cells were plated in 12-well plate and lysed by 100 μl of M-PER buffer supplemented with protease and phosphatase inhibitor cocktail at 4°C for 20 minutes. For Western Blots, the whole cell lysate was mixed with 5×SDS loading buffer thoroughly and denatured at 95°C for 5 minutes. The samples were subjected to the SDS-PAGE in Bio-Rad Mini-PROTEAN Tetra Cell and transferred by Thermo Scientific Owl VEP-2 (7351). The PVDF membrane was blocked with 5% NFDM (non-fat dried milk) at room temperature for 30 minutes and then incubated with the corresponding primary and HRP-conjugated secondary antibodies. Western blotting results were obtained by Bio-Rad *ChemiDoc*^™^ *XRS+ System* and Tanon *Fine-do* X6 (Shanghai, China). ImageJ software was used to for quantification. Mean values are shown in the Figures, and SDs are shown as error bars. All experiments were repeated for at least three times and representative results were shown in the Figures.

### CCK-8 assay for cell growth

The CCK-8 (Bestbio, China) assay was done according to the manufacturer’s instructions. Basically, HeLa-GDF cells plated in 96-well plate were treated with different concentrations of NH_4_Cl for 1, 2 or 3 days. The relative OD 450 nm value was used to evaluate the effect of NH_4_Cl on HeLa-GDF cells viability using CCK-8 assay by Bio-Rad iMark microplate reader.

### Ammonia quantification assay

Ammonia concentration was quantified by ammonia assay kit (abcam, 83360) according to the manufacturer’s instructions. Briefly, standard curve was prepared with NH_4_Cl standard solution. Then DMEM medium with Glutamine or GlutaMAX in different storage conditions was diluted with ammonia assay buffer for 20 times. Then the master mix for Ammonia Reaction Mix and a master mix for Background Reaction Mix were added to the standard and diluted sample medium to incubate at 37°C for 1 hour. The OD 570 nm value was acquired by SpectraMAX I3 (Molecular Devices) and used to calculate the ammonia concentration.

### Electron microscope analysis

CHO, CHO-GDF and HCT-116 cells were grown on plastic coverslips (Thermanox plastic coverslips, 174950) for 24 hours before NH_4_Cl treatment for 1 hour. Then the cells were fixed in 3% glutaraldehyde (containing 2% paraformaldehyde) at room temperature for 1 hour before they were placed at 4°C overnight and rinsed with PBS. The cells were post-fixed in PBS with 1% OsO_4_ and 1.5% potassium ferrocyanide at room temperature for 40 minutes twice and then exposed to 2% uranyl acetate in water. After PBS rinse, the cells were dehydrated in graded steps of ethanol and embedded in Spurr low viscosity media. Samples were polymerized at 70°C overnight. 100 nm ultra thin sections were cut onto copper grids by Leica Ultra CUT UC7. After post-stained with 0.2% lead citrate solution, the ultra thin sections were observed with 120KV electron microscope (Tecnai G2 Spirit BioTWIN, FEI company).

### Acridine Orange assay

HeLa cells were plated in 24-well plate 24 hours before experiment. Cells were treated with 5 μg/ml Acridine Orange (A3568, Life Technology) at 37°C for 15 minutes and washed with PBS for three times. To evaluate the effect of NH_4_Cl on vesicle pH, cells were treated with different concentrations of NH_4_Cl for 10 minutes before fluorescent microscope acquisition. Illuminated by 488-nm laser beam, the red fluorescence indicates acidity while the green fluorescence indicates alkalinity by I3 Channel equipped in Leica DMI4000B fluorescent microscope.

### Semi-quantitative RT-PCR

Total RNAs were isolated from harvested cells using RNAprep Pure kit (Tiangen), and 2 μg total RNA was used in each cDNA synthesis reaction using Maxima H Minus First Strand cDNA Synthesis Kit (Thermo Scientific) with an oligo (dT)_18_ primer. For GAPDH internal control RT-PCR, primers (GADPH-For, CCATCACCATCTTCCAGGAGCGAGATCC and GAPDH-Rev, GCCTGCTTCACCACCTTCTTGATGTC) were chosen from fully aligned sequence regions of human and Chinese hamster GAPDH genes so that both upstream and downstream primers have complete matches to both templates. RT-PCR reactions were performed with Premix PrimeSTAR HS (TaKaRa) according to manufacturer’s instructions. For DRD3 RT-PCR, nested PCR was performed. Briefly, two sets of primers, inside and outside primers, were selected from fully aligned human and Chinese hamster DRD3 gene sequences so that all primers (DRD3-out-For, TACCTGGAGGTGACAGGTGGAGTCTGG; DRD3-in-For, TGCTGTGATGTTTTTGTCACCCTGGATGTC; DRD3-in-Rev, GAAGGACACCACTGAAGAGTAGATGACAAAATCAGG; DRD3-out-Rev, GGCAGCCAGCAGACAATGAAGGC) have perfect matches to both sequences. For the primary PCR, 35 cycles were performed using the outside set of primers with the same PCR enzyme mix as described above. After that, one percent of the primary PCR products were used in the secondary PCR with the inside set of primers and amplified for 25 cycles.

### Statistical analysis

ImageJ software was used to quantify the protein relative levels shown by Western Blot and Graphpad prism 5 was used to analyze the data using One-way and Two-way ANOVA followed by Bonferroni post tests. P values < 0.05 were considered as statistically significant.

## Results

### Ammonia induces autophagy and mediates GFP-DRD3-Flag degradation

**Fig 1:**
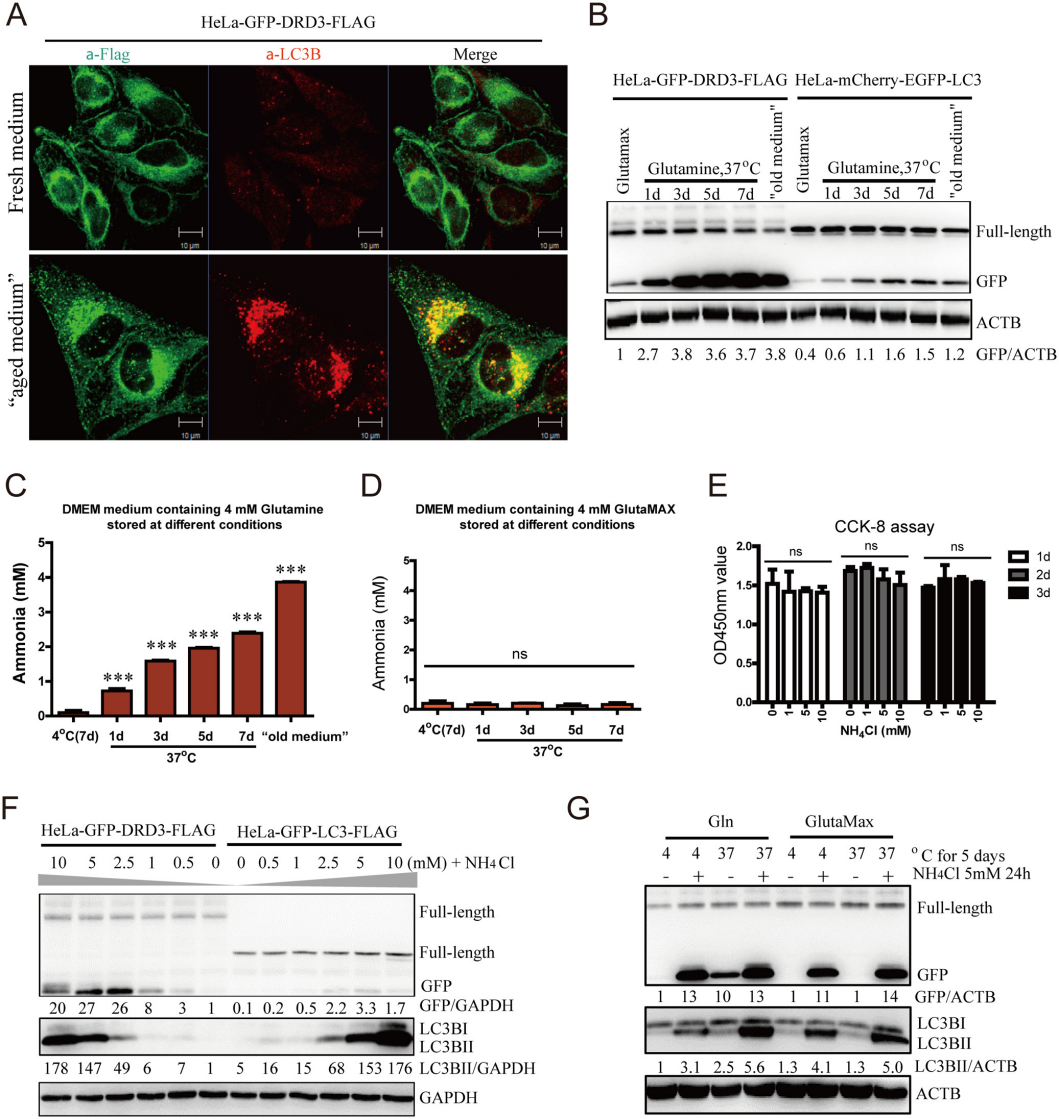
Aged medium and ammonia increase autophagy and cause GFP-DRD3-Flag degradation. (A) Immunofluorescence shows “aged medium” induces increased GFP-DRD3-Flag localization on autophagosomes. HeLa cells stably expressing GFP-DRD3-Flag were cultured in fresh DMEM medium containing Glutamine or in “aged medium”, which was cultured for a few days. Cells were fixed with -20°C methanol for 5 minutes and stained with anti-Flag or anti-LC3B antibodies. Scale bar, 10 μm. (B) “Old medium” induces free GFP fragment from GFP-DRD3-Flag. HeLa cells stably expressing GFP-DRD3-Flag cells or mCherry-EGFP-LC3 were incubated in culture medium containing Glutamine that had been incubated at 37°C for indicated times in the absence of cells (1–7 days). “Old medium” means a bottle of medium that was inappropriately stored during shipment (left out of refrigerator for at least 10 days). Representative Western blots show the effect of culture medium on GFP fragment from GFP-DRD3-Flag. (C) Cell culture medium containing Glutamine (medium alone, without cells) was stored at 4°C or 37°C for 1–7 days before they were tested for ammonia concentration. (D) Cell culture medium containing GlutaMax (medium alone, without cells) was stored at 4°C or 37°C for 1–7 days before they were tested for ammonia concentration. ***p<0.005. ns, not significant. (E) CCK-8 assays measure the effects of ammonia on cell vitality. HeLa cells stably expressing GFP-DRD3-Flag were cultured in the presence of 0, 1, 5 10 mM of ammonia for 1, 2 or 3 days before they were tested by CCK-8 assays. (F) Ammonia induces free GFP fragment from GFP-DRD3-Flag. HeLa cells stably expressing GFP-DRD3-Flag or GFP-LC3-Flag cells were cultured in different concentrations of NH_4_Cl for 24 hours. Representative Western blots are shown. (G) “Old medium” containing Glutamax does not induce free GFP fragment from GFP-DRD3-Flag unless ammonia was added. HeLa-GFP-DRD3-Flag cells were incubated in culture medium containing Glutamine or GlutaMAX that had been incubated in 37°C or 4°C for 5 days. NH_4_Cl was added for 24 hours before cells were harvested and processed for Western blots. Representative Western blots are shown. Densitometric analysis was performed and quantification results were labeled below the corresponding blots.

The Dopamine receptor D3 (DRD3) not only localizes to the plasma membrane but, like other GPCRs, is also internalized into intracellular vesicles. While using HeLa cells stably expressing GFP-DRD3-Flag to study the function of DRD3 in endocytic sorting and cytokinesis, we observed fewer cytoplasmic puncta of GFP-DRD3-Flag in cells 1 day after passage compared to 3 days or longer, which we termed “aged medium” (Fig 1A). The GFP-DRD3-Flag puncta induced in aged medium resembled autophagosomes induced by conditioned medium in previous studies. We stained for the autophagosome marker LC3B and found it colocalizes with GFP-DRD3-Flag, indicating that GFP-DRD3-Flag might enter autophagosomes for degradation (Fig 1A). In addition, storage of medium alone, without incubation with cells, at 37°C for one day or longer can significantly increase the degradation of GFP-DRD3-Flag and generate a free GFP fragment, an intermediate degradation product that resembles GFP-LC3 fragmentation upon autophagy perturbation (Fig 1B). We also tested a bottle of “old medium”, a batch of regular DMEM cell culture medium that has been inappropriately stored (without refrigeration for at least 10 days) during shipment. We found this “old medium” also significantly increased the degradation of GFP-DRD3-Flag and generate a free GFP fragment (Fig 1B).

Since we routinely used cell culture medium that contains Glutamine, we suspected the effect of aged medium might be due to breakdown of Glutamine into ammonia. To test this, we measured the ammonia concentration in the “old medium” and found that it is around 4 mM (Fig 1C), which is much higher than medium containing Glutamine and properly stored at 4°C for 7 days (around 0.1 mM ammonia, Fig 1C). In addition, we also tested ammonia concentration in medium containing Glutamine after they have been stored at 37°C for different days (Fig 1C). Our results show that the ammonia concentration is obviously increased after the medium was stored at 37°C for only one day, gradually increased over time and reached 2.5 mM after 7 days of 37°C storage (Fig 1C). In contrast, cell culture medium containing GlutaMAX (a non-degradable substitute for Glutamine) did not have increased ammonia even after 7 days of 37°C (Fig 1D). For Glutamine concentration, Sigma has shown that around 10% of Glutamine was degraded after 1 days of incubation at 37°C and around 70% of Glutamine was degraded after 7 days (https://www.sigmaaldrich.com/content/dam/sigma-aldrich/docs/Sigma/Product_Information_Sheet/t075.pdf).

To confirm the effect of ammonia on autophagy and GFP-DRD3-Flag degradation, we first tested the effect of ammonia on cell growth. We found that even 10 mM of ammonia for 3 days did not have significant inhibition on cell growth (Fig 1E). Then we tested its effect on GFP-DRD3-Flag degradation and autophagy. We added different concentrations of ammonium chloride (NH_4_Cl) to HeLa cells expressing GFP-DRD3-Flag or GFP-LC3-Flag and found that it induces GFP-DRD3-Flag degradation and generates a significant amount of free GFP fragment as well as increased LC3BII levels, indicating that the autophagosome number is increased (Fig 1F). In addition, we found that addition of ammonia as the free base or hydrochloride salt had the same effect on the GFP fragment and LC3B (S1 Fig) and the relative location of the GFP tag within the DRD3 construct or the presence of Flag tag did not affect GFP-DRD3 response to ammonia (S2 Fig). To reduce the possible variations caused by medium conditions, we used culture medium supplemented with GlutaMAX for the rest of this study. Aged medium containing GlutaMAX did not affect autophagosomes or cause GFP-DRD3-Flag degradation unless supplemented with ammonia (Fig 1G). In the meantime, to differentiate the effects were caused by ammonia by itself or pH change, we measured the medium pH with or without 5 mM NH_4_Cl and found that the pH was not affected after 5 mM NH_4_Cl addition. Therefore, ammonia was the reason for increased autophagosomes and DRD3 degradation in aged medium containing Glutamine.

### Ammonia has dual roles for autophagy

**Fig 2:**
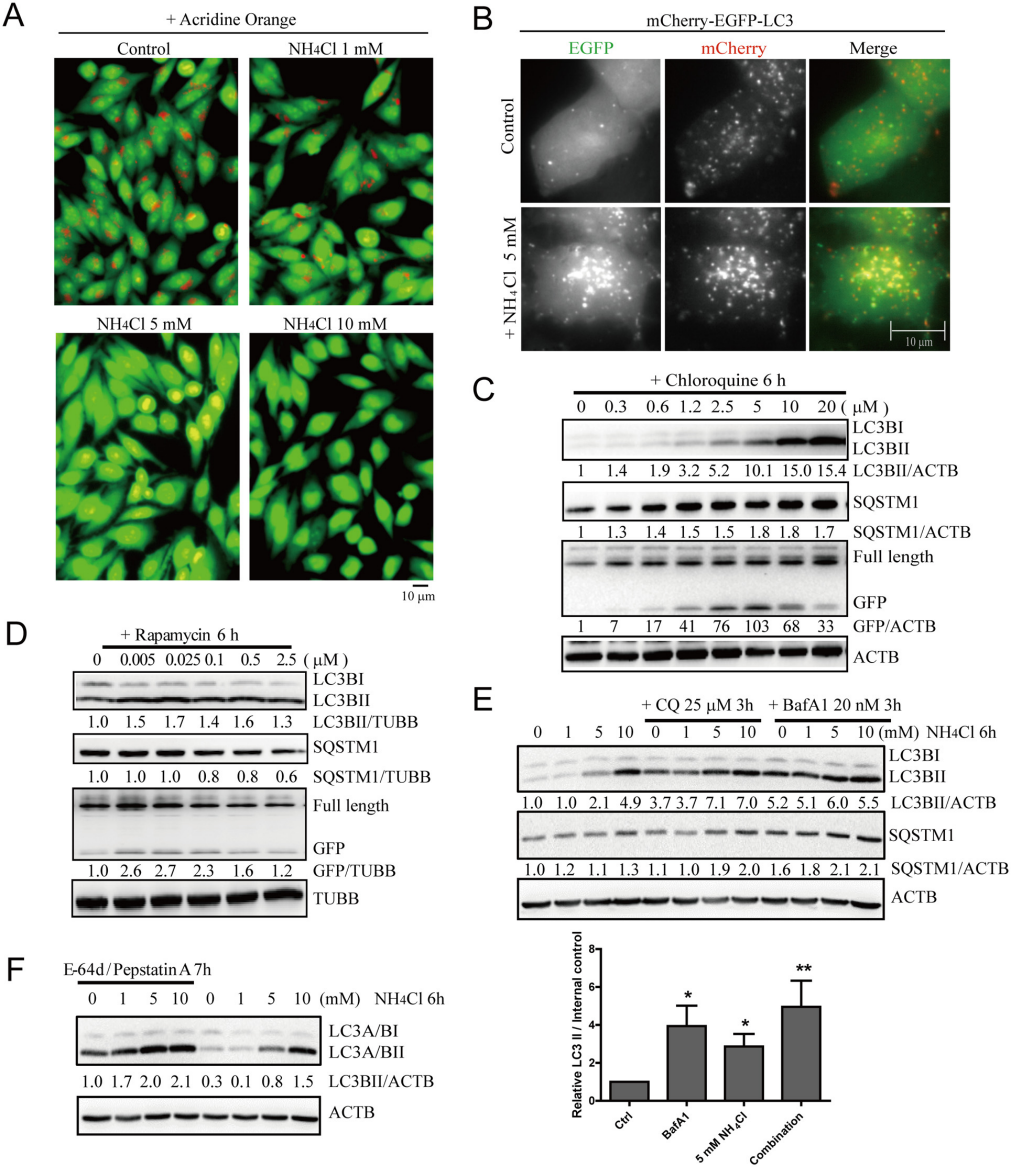
Ammonia has dual functions in autophagy regulation. (A) Acridine orange staining shows that 5–10 mM of NH_4_Cl increase the vesicular pH in cells. HeLa cells were treated with 5μg/ml Acridine Orange at 37°C for 15 minutes and washed with PBS. Then cells were treated with different concentrations of NH_4_Cl for 10 minutes before fluorescent microscope acquisition excited at 488 nm. (B) Live cell imaging shows ammonia increases both autophagosomes number and their pH. HeLa-mCherry-EGFP-LC3 cells were treated with 5 mM ammonium chloride for 2 hours before they were imaged. Scale bar, 10 μm. (C, D) Autophagy inhibitor increases SQSTM1/p62 while autophagy inducer decreases SQSTM1/p62. HeLa cells stably expressing GFP-DRD3-Flag were incubated with increasing concentrations of (C) Chloroquine, (D) Rapamycin, before cells were harvested and processed for Western blots. (E, F) NH_4_Cl induces autophagy. (E) HeLa cells stably expressing GFP-DRD3-Flag were incubated with increasing concentrations of NH_4_Cl for 6 hours with or without Chloroquine or Bafilomycin A1 for 3 hours. Representative Western blots are shown. Quantification of LC3BII/internal control from three independent experiments is shown on the bottom. *p<0.05, **p<0.01. (F) HeLa cells stably expressing GFP-DRD3-Flag were incubated with increasing concentrations of NH_4_Cl for 6 hours with or without E-64d/Pepstatin A for 7 hours. Representative Western blots are shown. Densitometric analysis was performed and quantification results were labeled below the corresponding blots.

The effect of ammonia on the autophagy pathway is complex. It was previously reported that ammonia can induce autophagy [5,11,12]. However, Ammonia is a weak base that can cross membranes in its unprotonated form, and neutralize acidic compartments. We used acridine orange, a dye that emits as red in acidic vesicles and green in non-acidic vesicles, to confirm that adding 5–10 mM of NH_4_Cl into the medium did increase the vesicular pH in cells (Fig 2A). By this mechanism it might in fact inhibit autophagy, which depends on acidification of the autophagosomes once it engulfs a target organelle. Therefore, it may have dual roles for autophagy. Since GFP (or EGFP) is pH-sensitive and its fluorescence is quenched in acidic vesicles while mCherry is pH-insensitive, a construct that expresses LC3 tagged with mCherry and EGFP in tandem (mCherry-EGFP-LC3) is a useful sensor to monitor autophagosomal pH changes in living cells. Ammonia increased both mCherry- and GFP-positive autophagosomes, which indicates that it increases both autophagosome number and their pH (Fig 2B).

To further clarify the role of ammonia, we took advantage of two markers: SQSTM1/p62 and LC3B. The difference between SQSTM1/p62 and LC3B as autophagy markers is that an increase in SQSTM1/p62 level usually indicates autophagic flux inhibition (serves as a cargo), while an LC3B increase can be either due to increased autophagy induction or autophagic flux inhibition (both a cargo and an autophagy machinery component). The autophagic flux inhibitors Chloroquine and Bafilomycin A1 both inhibit autophagosome acidification and cause LC3BII and SQSTM1/p62 accumulation (Fig 2C, S3A Fig), while autophagy inducers, such as dopamine or Rapamycin, increase LC3BII but decrease SQSTM1/p62 (Fig 2D). Since ammonia also increases the intra-vesicular pH to inhibit autophagic flux, adding NH_4_Cl alone does not decrease the overall amount of the autophagy cargo SQSTM1/p62 (Fig 2E). Its autophagy inducing role is usually masked by its role as an autophagic flux inhibitor so that the SQSTM1/p62 level is slightly increased. To confirm the inducer role of NH_4_Cl, we used Chloroquine and Bafilomycin A1 to accumulate autophagosomes in cells and add different concentrations of NH_4_Cl and found that LC3II was further increased, indicating increased autophagy induction by NH_4_Cl (Fig 2E). In addition, we also used E-64d/Pepstatin A to inhibit lysosomal proteases and found that they can also further accumulate LC3II, which confirmed the autophagy inducing role of ammonia (Fig 2F).

### PIK3C3/VPS34 plays vital roles in ammonia induced GFP-DRD3-Flag degradation and GFP fragment production

The free GFP fragment generated by GFP-LC3 is a characteristic intermediate degradation product along autophagic flux so that it is dynamically controlled by different concentrations of autophagic flux inhibitors [41]. We believe that the GFP fragment of GFP-DRD3-Flag is generated in a similar way (S3B Fig). GFP protein is tightly folded and therefore very stable, which makes it less susceptible to degradation along the autophagic flux than its partner proteins LC3 or DRD3. Autophagy blockers such as Bafilomycin A1 at lower concentrations will block autophagic flux and GFP degradation, which cause GFP fragment accumulation (S3A Fig). However, when they are used at higher concentrations, autophagic flux is further blocked, which prevents the whole protein (GFP-LC3 and GFP-DRD3) degradation through autophagic flux. In this case, the GFP fragments disappear because they remain intact together with LC3 and DRD3 as full length GFP-LC3 and GFP-DRD3 protein (S3A Fig). This concentration-dependent GFP fragment generation resembles GFP-LC3, which is commonly used as an autophagic flux marker [22,41,42].

Our data suggest that ammonia not only serves as an inducer for autophagy, but also acts as a ligand to induce DRD3 internalization and degradation. It is clear that ammonia can induce significantly more GFP fragment from GFP-DRD3 than from GFP-LC3 (Fig 1B and 1C). In addition, a general autophagy inducer, such as the MTOR inhibitor Rapamycin, does not significantly increase GFP-DRD3-Flag on autophagic structures nor generate free GFP fragment (S3C and S3D Fig). We reason that DRD3 is not only an ammonia sensor but also is recruited to autophagosomes for degradation upon ammonia stimulation.

**Fig 3:**
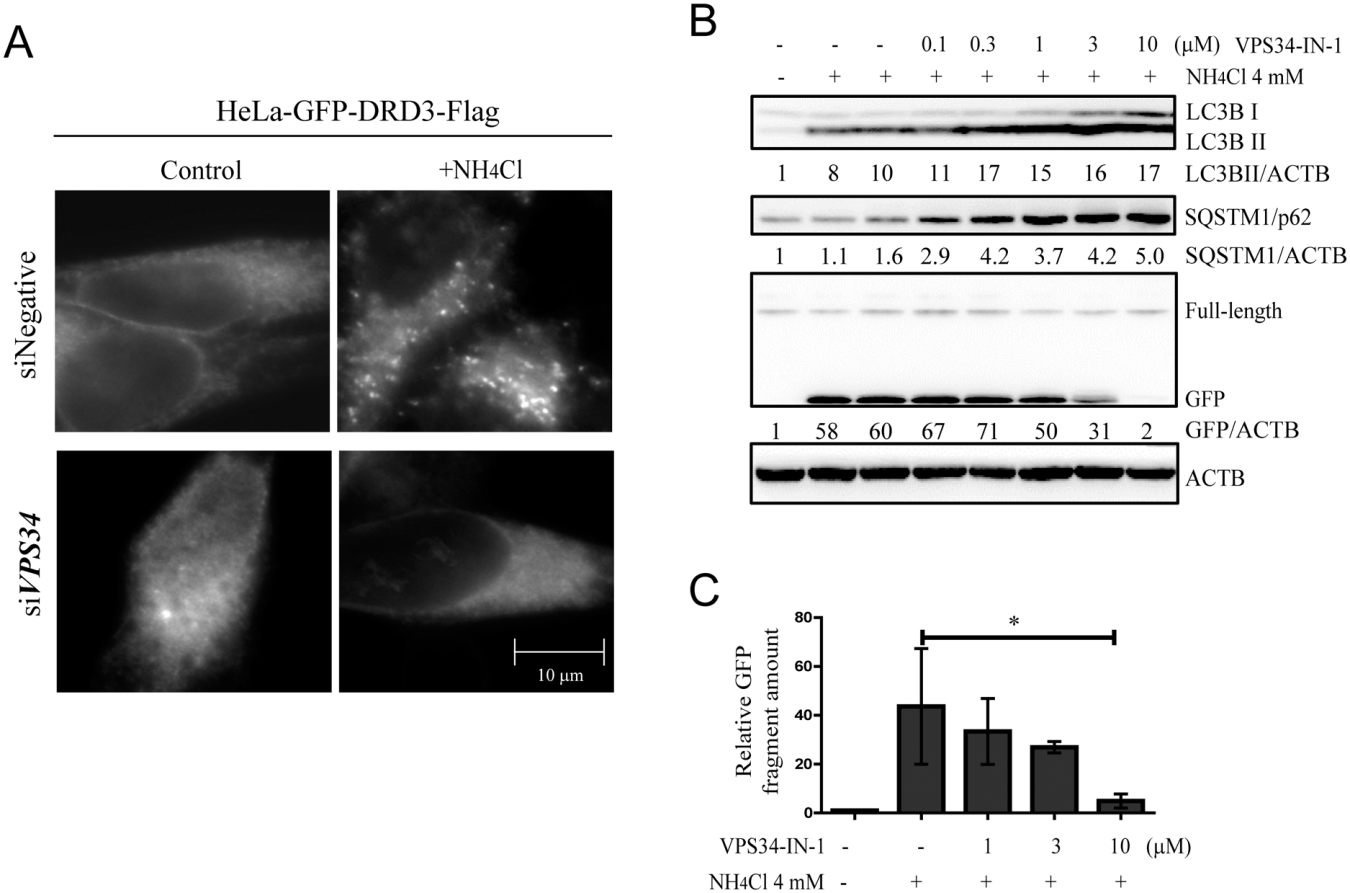
PIK3C3/VPS34 plays important roles in ammonia-induced GFP-DRD3-Flag degradation and GFP fragment accumulation. (A) HeLa-GFP-DRD3-Flag cells were transfected with 80 nM Negative or *PIK3C3/VPS34* siRNAs for 72 hours and treated with 10 mM NH_4_Cl for 24 hours before they were fixed and stained with anti-Flag antibodies. Scale bar: 10 μm. (B) Inhibition of PIK3C3/VPS34 prevents free GFP fragment generation from GFP-DRD3-Flag. HeLa-GFP-DRD3-Flag cells were treated with NH_4_Cl and VPS34-IN1 for 24 hours before cells were harvested and processed for Western blots. Representative Western blots are shown. (C) Quantification of relative GFP fragment amount in (B). Data show mean ± s.d. n = 3. *p<0.05. Representative Western blots are shown. Densitometric analysis was performed and quantification results were labeled below the corresponding blots.

Next we examine the role of autophagy in ammonia-induced GFP-DRD3-Flag degradation. Immunofluorescence experiments show that *PIK3C3/VPS34* RNAi efficiently reduced ammonia -induced GFP-DRD3-Flag puncta (Fig 3A). It was shown that inhibition of PIK3C3/VPS34 by a specific inhibitor VPS34-IN1 [39] can efficiently prevent both starvation and MTOR inhibitor-induced autophagy [43] because PIK3C3/VPS34 is required for both initiation and progression of autophagy. We used VPS34-IN1 and found that it could significantly reduce GFP fragment (Fig 3B and 3C). These experiments demonstrate that PIK3C3/VPS34 plays an important role in ammonia-induced GFP-DRD3-Flag degradation. It should be noted that PIK3C3/VPS34 also has function in membrane trafficking so it is possible that the endosome pathway is also involved in ammonia-induced DRD3 degradation. Therefore, GFP-DRD3-Flag degradation induced by ammonia seems to involve PIK3C3/VPS34-dependent pathways.

### Ammonia specifically enriches DRD3 onto autophagosomes

**Fig 4:**
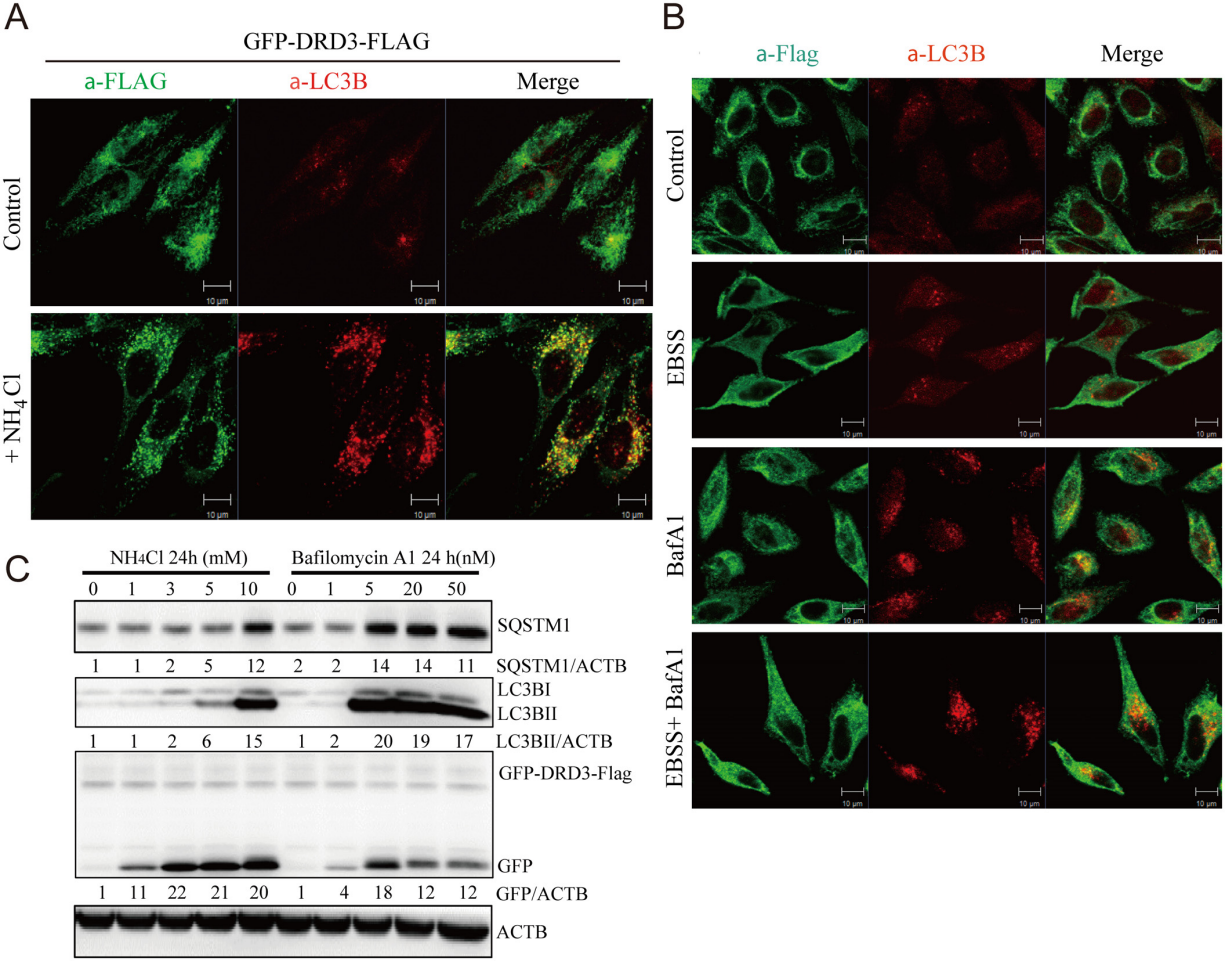
Ammonia, but not starvation or Bafilomycin A1, enriches DRD3 on autophagosomes. (A) NH_4_Cl induces DRD3 enrichment on autophagosomes. HeLa cells stably expressing GFP-DRD3-FLAG were incubated in control medium or in medium containing 10 mM NH_4_Cl for 24 hours before they were fixed and stained with anti-Flag (green) and anti-LC3B (red). The inset shows the enlarged view of the corresponding boxed regions in NH_4_Cl-treated cells. Scale bar, 10 μm. (B) HeLa cells stably expressing GFP-DRD3-FLAG were treated with Bafilomycin A1, EBSS or EBSS combined with Bafilomycin A1 for 2 hours before fixing with -20°C methanol and staining with anti-Flag or anti-LC3B antibodies. Scale bar, 10 μm. (C) Bafilomycin A1 induces much less GFP fragment from GFP-DRD3-Flag than NH_4_Cl does. HeLa cells stably expressing GFP-DRD3-Flag were treated with different concentrations of NH_4_Cl or Bafilomycin A1 for 24 hours. Cells were harvested for Western blots. Representative results are shown in the Figures. Densitometric analysis was performed and quantification results were labeled below the corresponding blots.

As a membrane protein, GFP-DRD3-Flag is observed to have some membrane staining and mostly diffuse internal staining. However, upon NH_4_Cl addition, most GFP-DRD3-Flag re-localized to autophagic structures (Fig 4A), which indicates that DRD3 is likely an autophagic cargo that enters the autophagy pathway upon NH_4_Cl stimulation. It is clear that starvation, Bafilomycin A1 or starvation combined with Bafilomycin A1 do not increase DRD3 on autophagic structures (Fig 4B). In addition, Ammonia also produces more GFP fragment from GFP-DRD3 than Bafilomycin A1 does (Fig 4C). These results show that co-localization of DRD3 with autophagosomes is specifically induced by aged medium and ammonia.

**Fig 5:**
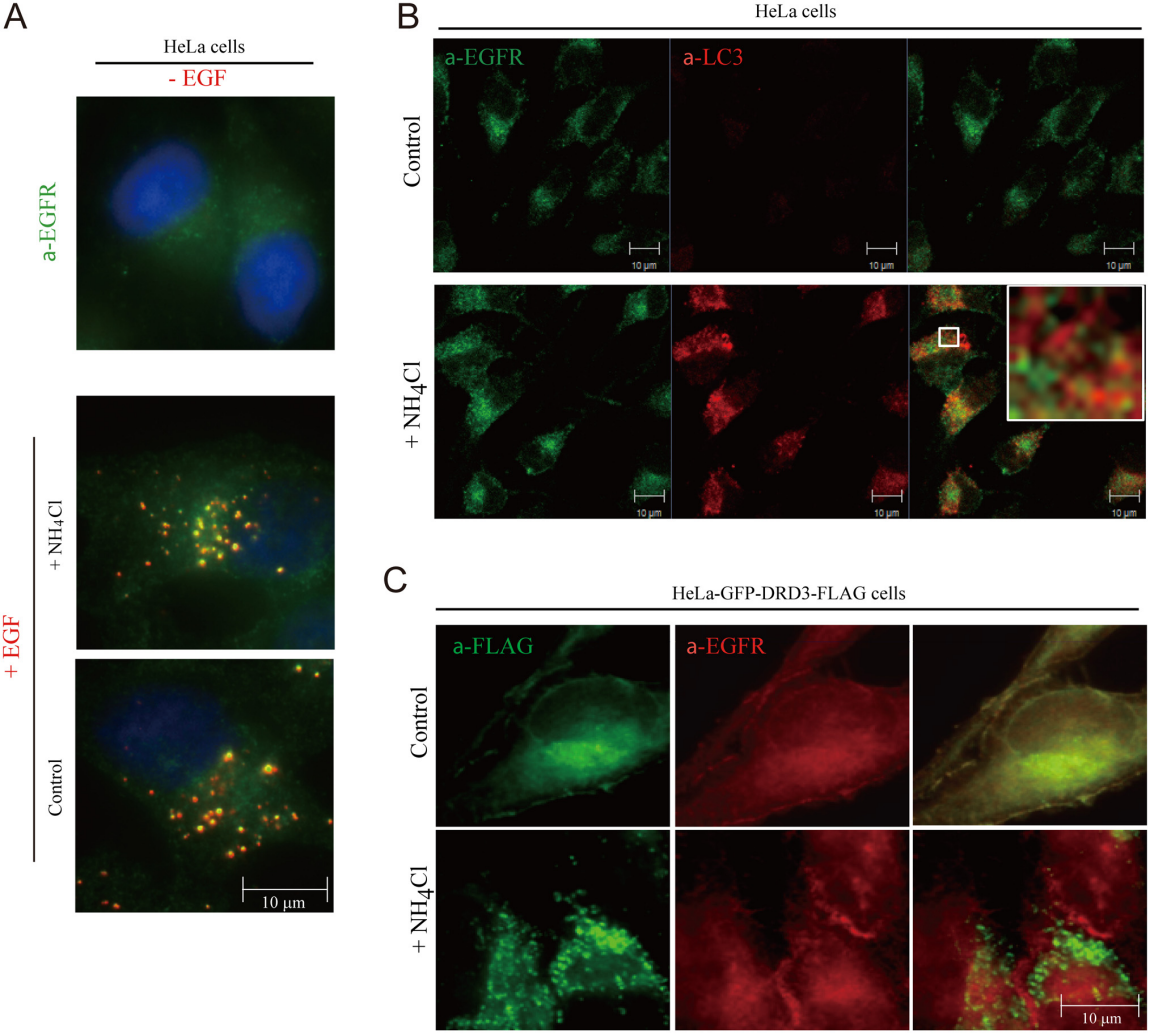
Ammonia does not enrich membrane protein EGFR on autophagosomes. (A) EGFR internalizes upon EGF addition. HeLa cells were treated with or without 10 mM NH_4_Cl for 24 hours before they were pulsed and chased with EGF-Alexa 555 (red). Cells were fixed in 4% formaldehyde and stained with anti-EGFR antibody (green). (B) NH_4_Cl does not induce EGFR enrichment on autophagosomes. HeLa cells were treated with or without 10 mM NH_4_Cl for 24 hours before they were fixed in -20°C methanol and stained with anti-EGFR (green) and LC3 (red) antibodies. Representative images are shown. The inset shows the enlarged view of the corresponding boxed region in NH_4_Cl-treated cell. (C) HeLa cells stably expressing GFP-DRD3-FLAG were incubated in control medium or in medium containing 10 mM NH_4_Cl for 24 hours before they were fixed in -20°C methanol and stained with anti-Flag (green) and anti-EGFR (red) antibodies. Scale bar, 10 μm.

Movement of DRD3 from the plasma membrane to autophagosomes in response to ammonia could be a general effect on membrane trafficking, or a specific effect on this receptor. To distinguish these possibilities, we imaged another plasma membrane receptor, EGFR (a receptor tyrosine kinase), which internalizes upon stimulation by its ligand, EGF and is then degraded in lysosomes (Fig 5A). We found that EGFR did not colocalize with LC3 (Fig 5B) or DRD3 (Fig 5C) upon NH_4_Cl treatment. These results indicate that ammonia-induced GFP-DRD3-Flag aggregation on autophagosomes is not general to membrane proteins. Thus, the effect of ammonia appears to be specific for DRD3.

### DRD3 serves as an ammonia sensor in cell membrane

**Fig 6:**
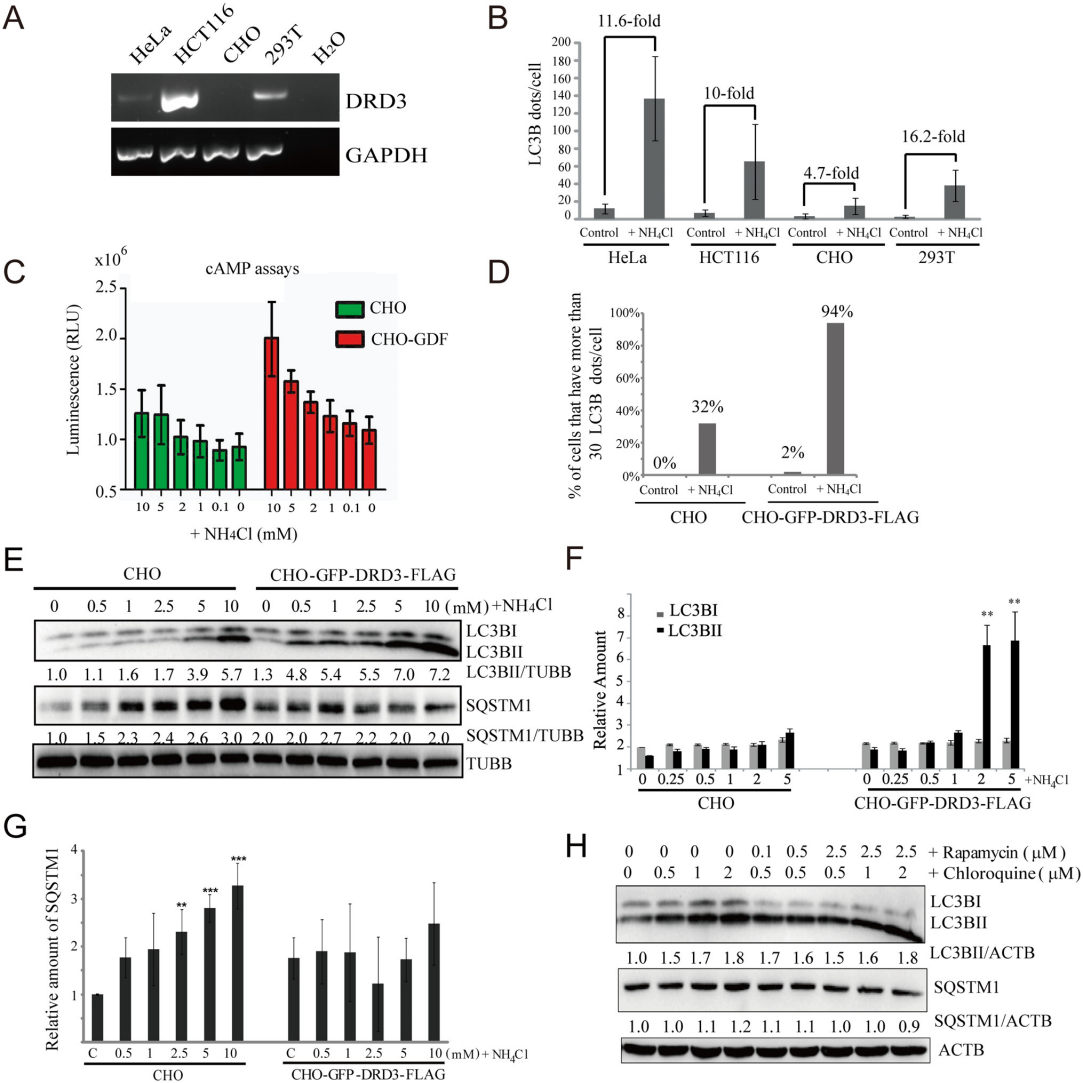
DRD3 is an ammonia sensor. (A) Semi-quantitative Reverse-Transcription PCR to compare the relative mRNA level in HeLa, HCT116, CHO and 293T cells. Last lane shows RT-PCR result using ddH_2_O as template. Human and Chinese Hamster DRD3 and GAPDH sequences were aligned to design primers that match DRD3 and GAPDH in both human and Chinese Hamster. (B) Different cells have different autophagy responses to ammonia. HeLa, HCT116, CHO and 293T cells were treated with 5 mM NH_4_Cl for 24 hours before they were fixed and stained with anti-LC3B antibody. Quantification of LC3B puncta in HeLa, HCT116, CHO and 293T cells are shown. Fifty cells were counted for LC3B puncta in three independent experiments. (C) Transfected GFP-DRD3-Flag responds to ammonia in a traditional GPCR assay. cAMP-Glo experiment using CHO, or CHO cells that stably express GFP-DRD3-Flag to measure the effects of ammonia on cAMP levels in cells. (D) Quantification of LC3 puncta in CHO, or CHO cells that stably express GFP-DRD3-Flag before and after NH_4_Cl treatment. Fifty cells were counted for LC3B puncta. n = 3. (E) Transfected GFP-DRD3-Flag increases autophagy response to ammonia. Representative Western blots of CHO, or CHO cells that stably express GFP-DRD3-Flag treated with different concentrations of NH_4_Cl for 24 hours. (F) Quantification of LC3BI and LC3BII in (E) from three independent results. Data were all normalized to LC3BII control in CHO cells. (G) Quantification of the relative level of SQSTM1/p62 in (E) from three independent results. Data were all normalized to control in CHO cells. For (F) and (G), **p<0.01, ***p<0.005. Data show mean ± s.d. n = 3. (H) Adding both autophagy inducer and inhibitor counteracts on SQSTM1/p62. HeLa cells stably expressing GFP-DRD3-Flag cells were incubated with increasing concentrations of Chloroquine and/or Rapamycin for 6 hours. Representative Western blots are shown. Densitometric analysis was performed and quantification results were labeled below the corresponding blots.

To test if DRD3 is also a receptor for the ammonia induced autophagy response, we sought cell lines lacking DRD3. Previous reports have shown that CHO cells do not express dopamine receptors [44]. To confirm this, we compared the DRD3 level in a few different cell lines. Since the commercially available antibodies for DRD3 do not reliably work in Western blot or immunofluorescence, we designed primers to match both human and Chinese hamster DRD3 and GAPDH sequences and did a semi-quantitative RT-PCR (reverse transcription-PCR) to check the DRD3 level in these cell lines. Our results confirm that CHO cells do not express DRD3 but HeLa, HCT116 and 293T do (Fig 6A). We found that CHO cells showed much weaker activation of autophagy by ammonia than three other cell lines we tested (Fig 6B). To explore the involvement of DRD3 in sensing ammonia and causing this difference in these cell lines, we stably overexpressed GFP-DRD3-FLAG in CHO cells and compared to non-expressing CHO cells for their responses to ammonia. Since DRD3 is a GPCR that will induce cellular cAMP decrease upon agonist stimulation, we used a traditional cAMP assay to examine its function. We found that ammonia induces a dose-dependent cAMP decrease in CHO-GFP-DRD3-Flag cells (Fig 6C), indicating that overexpressed DRD3 is functional and that ammonia is a DRD3 agonist for its traditional downstream cAMP pathway. In addition, we found that LC3B puncta (Fig 6D) and LC3BII levels (Fig 6E and 6F) were much more induced by ammonia in the CHO-GFP-DRD3-Flag cell line relative to untransfected CHO cells, supporting our hypothesis that DRD3 is an ammonia sensor upstream of autophagy.

Since ammonia has dual roles in autophagy and its inducer role is related to DRD3, we observed differential effects of ammonia in untransfected CHO cells (do not express DRD3) with CHO cells stably transfected with GFP-DRD3-Flag. In CHO cells lacking DRD3, NH_4_Cl mainly acts as an autophagic flux inhibitor so that it increases both LC3BII and SQSTM1/p62 (Fig 6E and 6G, left). However, in CHO cells overexpressing GFP-DRD3-Flag, the dual roles of NH_4_Cl as both inducer and inhibitor lead to balanced SQSTM1/p62 levels (Fig 6E and 6G, right). This is because a simultaneous increase of autophagy induction and a block of late-stage autophagic flux will result in an even greater increase in LC3BII, but not SQSTM1/p62 because the autophagy induction causes SQSTM1/p62 degradation while autophagic flux inhibition causes SQSTM1/p62 accumulation. The number of autophagosomes is usually inversely correlated with SQSTM1/p62 level. For example, both Rapamycin and starvation treatment increase autophagosomes but decrease SQSTM1/p62 levels due to accelerated SQSTM1/p62 degradation. Consistent with this rationale, the combination of an autophagy inhibitor, Chloroquine, with an inducer, Rapamycin, led to decreased SQSTM1/p62 compared to Chloroquine alone because Rapamycin can increase SQSTM1/p62 degradation (Fig 6H). The above results confirm the dual roles of ammonia in autophagy. Ammonia works as an inducer through DRD3, while as an autophagic flux inhibitor it inhibits intra-vesicular pH in cells.

### Ammonia induced autophagy by MTOR is mediated by DRD3

**Fig 7:**
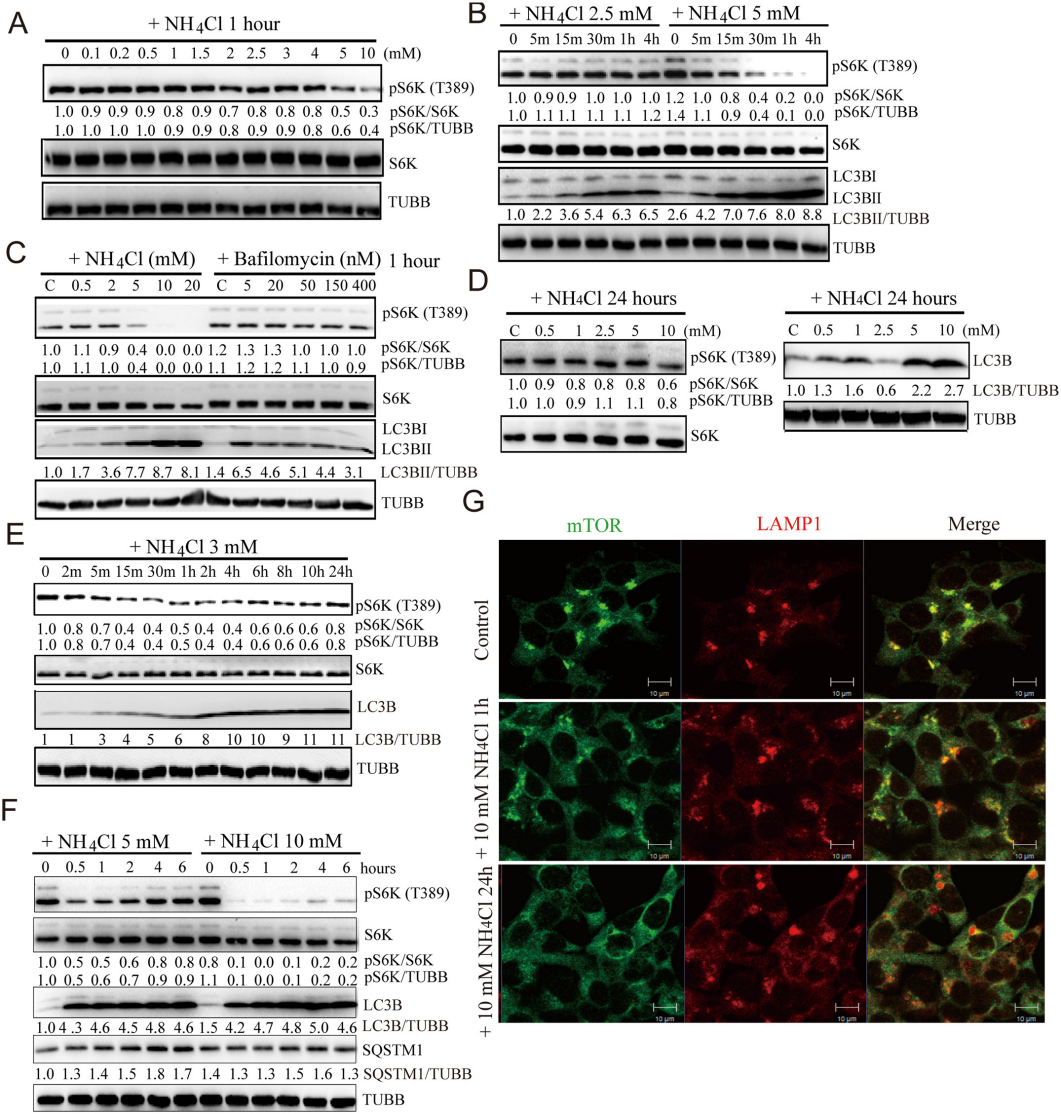
Ammonia inhibits MTOR activity and affects its localization. (A-F) Ammonia inhibits MTOR activity in cells. (A) Representative Western blots analyze MTOR activity in HeLa cells stably expressing GFP-DRD3-Flag treated with different concentrations of ammonia for 1h. (B) Representative Western blots analyze MTOR activity in HeLa cells stably expressing GFP-DRD3-Flag treated with 2.5 mM or 5 mM ammonia for 5 min to 4 h. (C) Representative Western blots analyze MTOR activity in HeLa cells stably expressing GFP-DRD3-Flag treated with different concentrations of ammonia or Bafilomycin A1 for 1 h. (D) Representative Western blots analyze MTOR activity in HeLa cells stably expressing GFP-DRD3-Flag treated with different concentrations of ammonia for 24 h. (E) Representative Western blots analyze MTOR activity in HeLa cells stably expressing GFP-DRD3-Flag treated with 3 mM ammonia for 2 min to 24 h. (F) Representative Western blots analyze MTOR activity in HeLa cells stably expressing GFP-DRD3-Flag treated with 5 mM or 10 mM ammonia for 30 min to 6 h. (G) Ammonia reduces MTOR localization on lysosomes. Immunofluorescence of HCT116 cells treated with 10 mM NH_4_Cl for 1 hour or 24 hours. Cells were fixed with 4% formaldehyde at room temperature for 20 minutes and immunostained with anti-MTOR and anti-LAMP1(H4A3) antibodies. Scale bar, 10 μm. n = 3. Representative Western blots are shown. Densitometric analysis was performed and quantification results were labeled below the corresponding blots.

Since MTORC1 plays a critical role in autophagy induction and is the key player in T1R1 and T1R3-mediated autophagy, we first examined whether ammonia affects MTORC1 activity in cells. We treated with ammonia for one hour compared to the longer incubation time above (and in the previous two studies for ammonia induced autophagy) [5,11] to reduce complexity and potential artifacts in the complicated signal transduction networks after prolonged treatment. We found that 5 and 10 mM of ammonia were able to inhibit the phosphorylation of S6K (T389), a major substrate of MTORC1 (Fig 7A and 7B). To test if the MTOR effects we observed were simply due to blocking acidification, we compared ammonia treatment with Bafilomycin A1, a widely used V-ATPase inhibitor that efficiently inhibits vesicle acidification. We found that phospho-S6K (T389) was not much affected by Bafilomycin A1 (Fig 7C). In untransfected HeLa and HCT116 cells, phospho-S6K (T389) was also inhibited by ammonia (S4A Fig). These data demonstrate that MTOR is involved in ammonia-induced autophagy.

Due to the complexity of MTOR related signaling pathway in cells, it is not surprising that inhibition of ammonia-induced MTOR activity is not only concentration-dependent but also time-dependent. We tested different concentrations of ammonia at 24 hours and found that the pS6K inhibition is not as obvious as after 1 hour (Fig 7D). Using fixed concentrations for a series of different time points, we found that the pS6K is decreased at shorter time points but was reactivated after longer incubation (Fig 7E and 7F), probably due to feedback loops of related pathways in cells after prolonged treatment.

To test how MTOR is regulated by ammonia, first we wanted to test if ammonia had a direct effect on MTOR kinase activity. We performed *in vitro* kinase assays using the MTORC1 complex co-immunoprecipitated with anti-RAPTOR antibody from the lysate of Raptor over-expressing HEK293T cells and purified EIF4EBP1 as substrate [40]. Our results show that ammonia had no effect on MTOR kinase activity *in vitro* (S4B Fig), indicating that ammonia affects MTOR indirectly, which is consistent with our hypothesis that DRD3 is the mediator. Next we examined the ammonia’s effect on MTOR localization because MTOR is recruited to lysosomes to regulate autophagy [45]. We found that ammonia decreased MTOR localization on lysosomes (Fig 7G).

**Fig 8:**
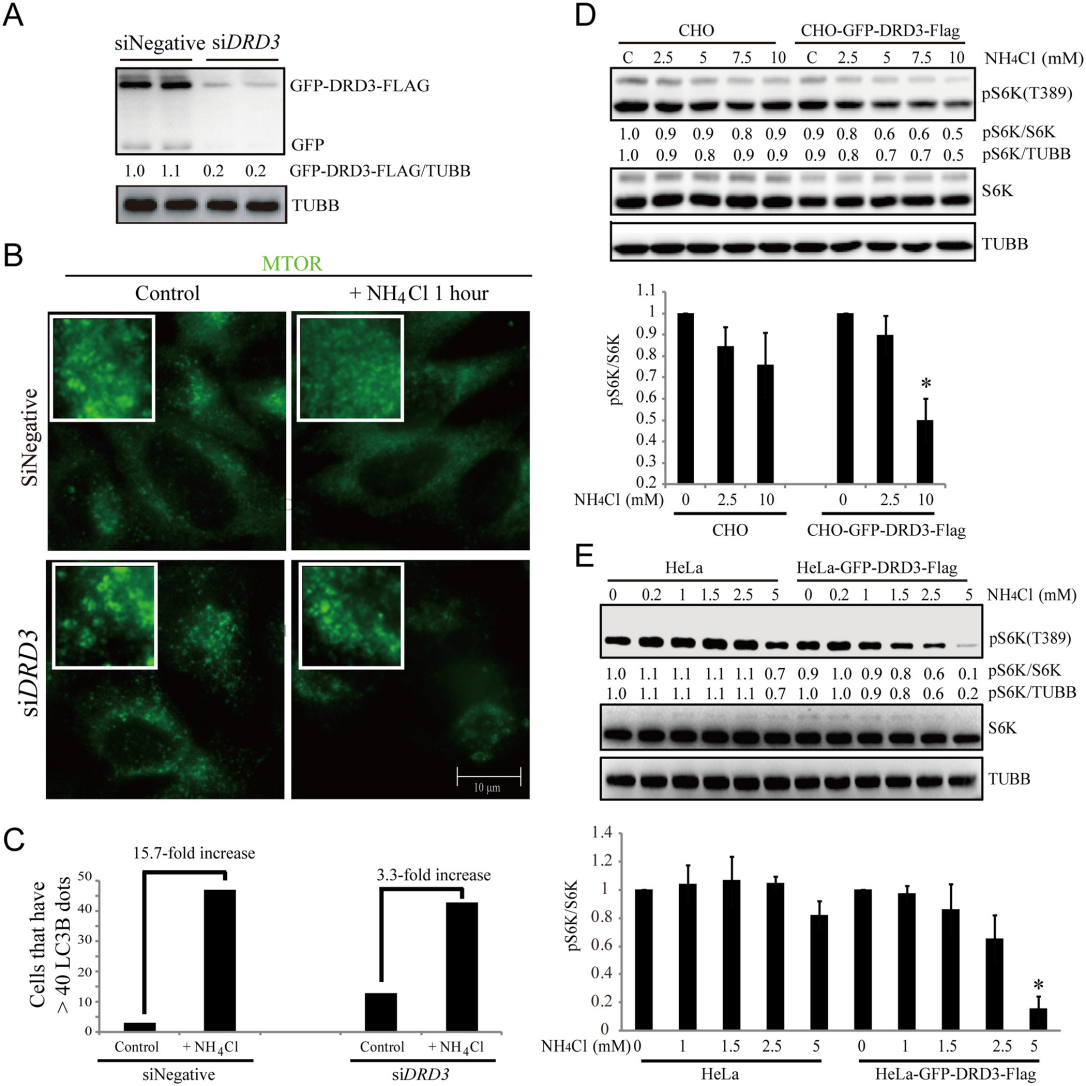
DRD3 mediates ammonia-induced MTOR localization change and autophagy induction. (A) Representative Western blots show that DRD3 RNAi efficiently knockdown DRD3. HeLa cells stably expressing GFP-DRD3-Flag were treated with 40 nM Negative or *DRD3* siRNA for 3 days. Samples were loaded in duplicate for verification. Anti-GFP and anti-Tubulin were used. (B) Immunofluorescence shows that DRD3 RNAi abolishes ammonia-induced MTOR localization changes. HeLa cells were incubated with 40 nM Negative or *DRD3* siRNA for 3 days before they were treated with 10 mM NH_4_Cl for 1 hour. Cells were fixed and stained with anti-MTOR. Scale bar, 10 μm. (C) Quantification of LC3B dots in HeLa cells treated with Negative or *DRD3* siRNA and treated with 10 mM NH_4_Cl for 24 hours. Fifty cells were counted for their LC3B dots. n = 3. (D, E) DRD3 mediates ammonia-induced MTOR activity inhibition. (D) CHO versus CHO-GFP-DRD3-Flag cells were treated with NH_4_Cl for 0.5–1 hour before they were harvested for Western blots using anti-pS6K, anti-S6K and anti-Tubulin antibodies. Quantifications show the relative value of pS6K/S6K, normalized to CHO cells with no ammonia addition. Data show mean ± s.d. n = 3. (E) HeLa versus HeLa-GFP-DRD3-Flag cells were treated with different concentrations of NH_4_Cl for 0.5–1 hour before they were harvested for Western blots using anti-pS6K, anti-S6K and anti-Tubulin antibodies. Quantifications show the relative value of pS6K/S6K, normalized to HeLa cells with no ammonia addition. Data show mean ± s.d. n = 3. Representative Western blots are shown. Densitometric analysis was performed and quantification results were labeled below the corresponding blots.

Next, we examined whether DRD3 actually mediates ammonia-induced MTOR localization changes. Using RNAi experiment (Fig 8A), we found that upon ammonia treatment for one hour, the vesicular localization of MTOR was decreased in control cells but not much affected in DRD3 RNAi knockdown cells (Fig 8B). In addition, we also observed that an increase in cytoplasmic LC3B dots caused by ammonia is reduced five-fold in DRD3 RNAi cells (Fig 8C). We also compared ammonia-induced S6K phosphorylation changes in CHO cells versus CHO-GFP-DRD3-Flag cells (Fig 8D), as well as in HeLa versus HeLa-GFP-DRD3-Flag cells (Fig 8E). We found that S6K phosphorylation is not much affected by ammonia in CHO cells but is reduced in CHO-GFP-DRD3-Flag cells (Fig 8D). In addition, ammonia-induced S6K phosphorylation changes are much more obvious in HeLa-GFP-DRD3-Flag cells compared to HeLa cells (Fig 8E). These data all confirm the participation of DRD3 in bridging MTOR and ammonia sensing.

**Fig 9:**
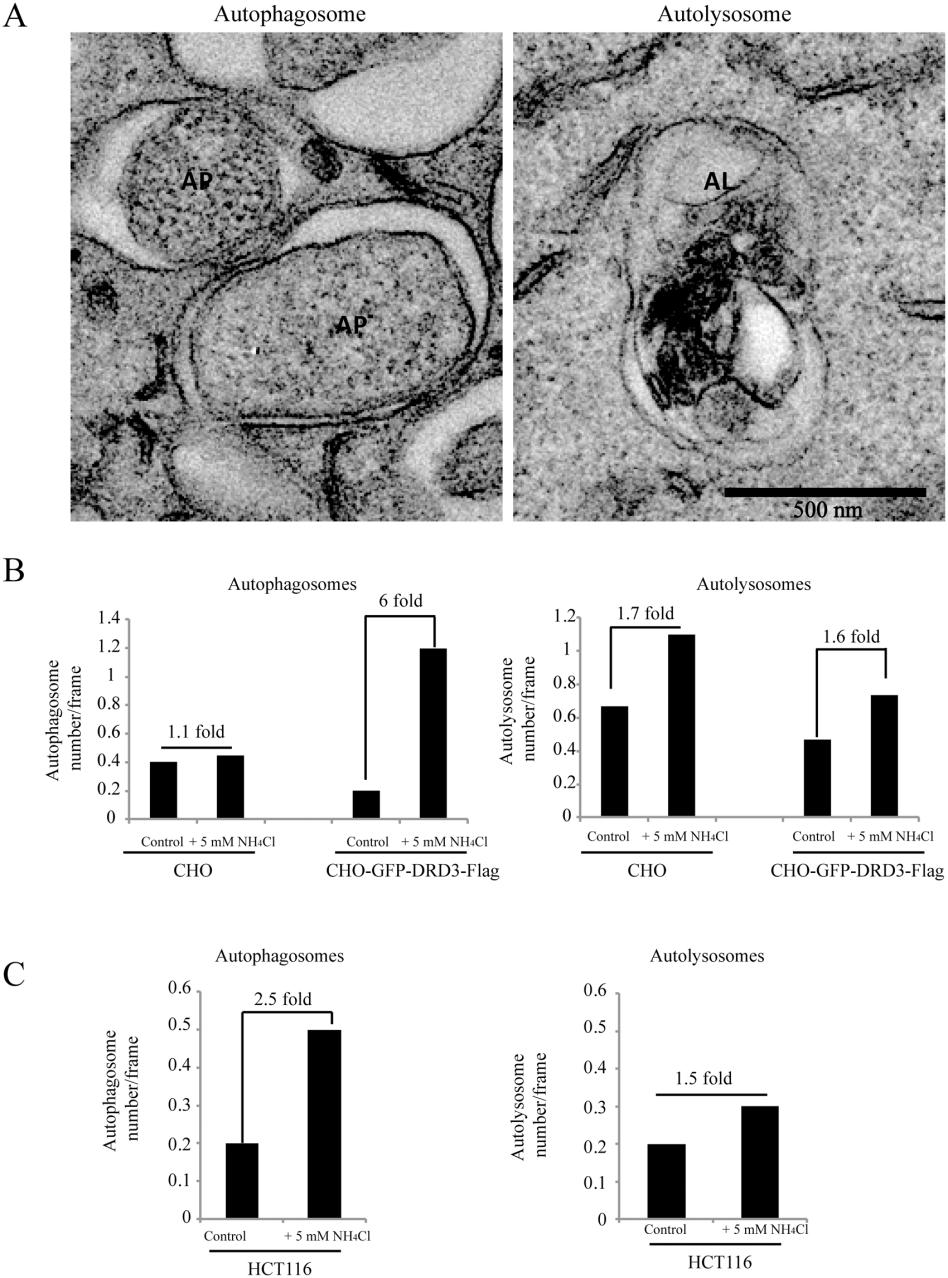
Electron Microscopy of autophagosomes and autolysosomes in cells expressing different levels of DRD3. (A) Representative electron microscopic images of two autophagosomes (AP, left panel) and an autolysosome (AL, right panel). Scale bar: 500 nm. (B) Quantification of autophagosomes and autolysosomes in CHO cells and CHO cells overexpressing GFP-DRD3-Flag. (C) Quantification of autophagosomes and autolysosomes in HCT116 cells. Results represent mean value of 15–25 random cell images per experimental group.

**Fig 10:**
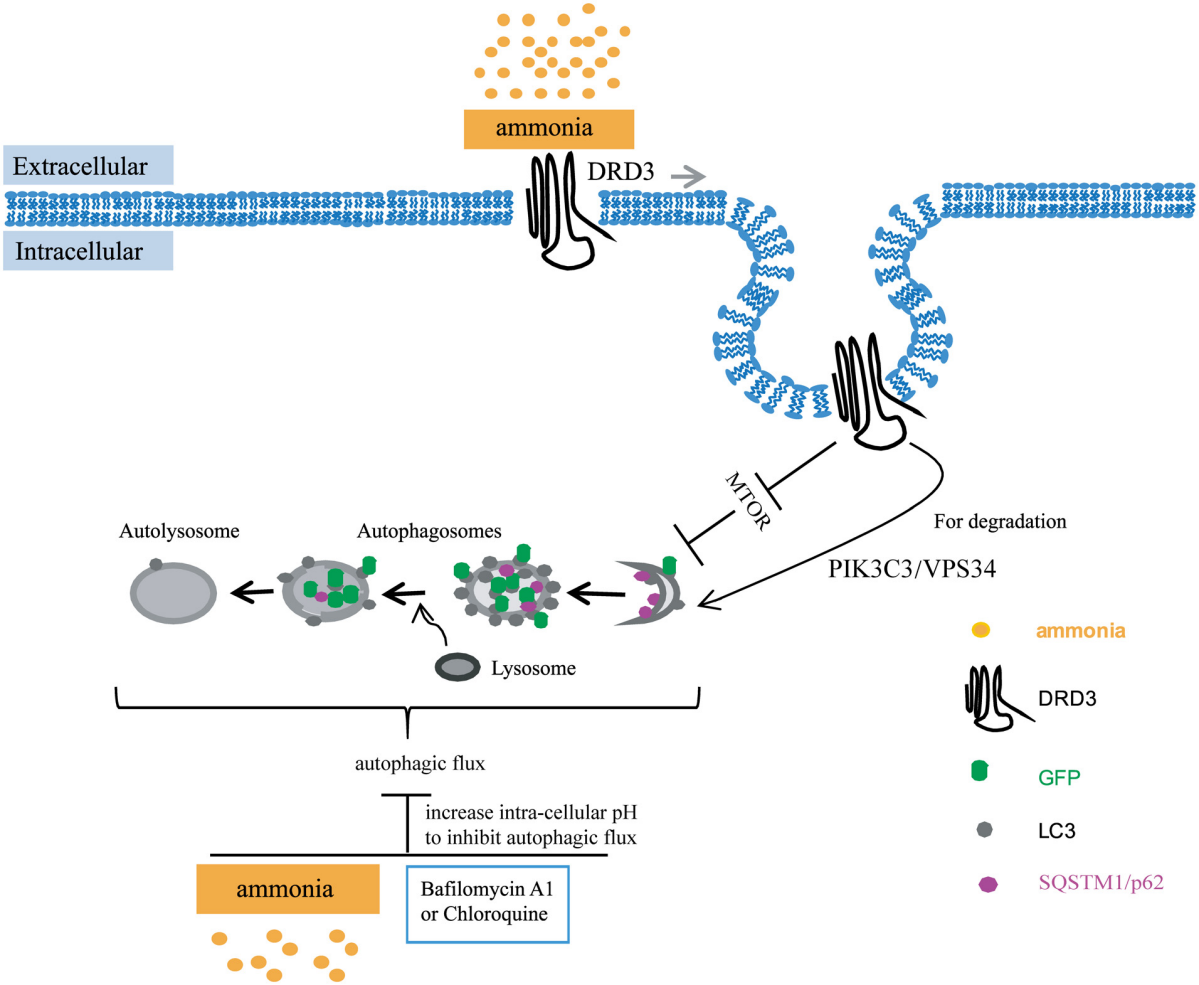
A model illustrates the roles of ammonia in autophagy regulation. Our data show that ammonia has dual roles in autophagy. One is to induce autophagy through Dopamine receptor D3 and MTOR, the other is to inhibit autophagic flux by perturbing vesicle pH. At the same time, it also enriches DRD3 on autophagosomes for degradation.

To get more confirmative information of ammonia induced autophagic structures in cells expressing different levels of DRD3, we did the EM experiments for CHO cells transfected with or without GFP-DRD3-Flag as well as HCT116 cells, which has high level of DRD3 expression. We compared the autophagosomes and autolysosomes number with or without 5 mM of ammonia for 3 hours (Fig 9). We counted both autophagosomes and autolysosomes. It is clear that ammonia induces much more autophagosomes in cells with higher level of DRD3 (HeLa-GFP-DRD3-Flag and HCT116 cells) than in CHO cells, which do not express DRD3. These results confirmed the involvement of DRD3 in ammonia-induced autophagy (Fig 10).

## Discussion

Cells and organisms need to sense extracellular cues such as growth factors, amino acids as well as other nutrients, so that they can coordinate their growth, development and metabolism accordingly. Here, we identify the dopamine receptor D3 as an ammonia sensor. At the same time, it is also clear that ammonia is not the only autophagy stimulus that DRD3 can sense. Some studies show that Dopamine receptor ligands can affect autophagy. For example, dopamine can increase LC3BII in neuroblastoma [46]; sertindole, an inhibitor of dopamine receptor D2 (DRD2) can induce autophagy in neuroblastoma [47]; raclopride, an inhibitor of DRD2 can induce autophagy in cardiacmyocytes [48]; N-methyl-4-phenylpyridinium (MPP+), a dopaminergic neurotoxin increases LC3B and decreases SQSTM1/p62 [49]. These all indicate that dopamine receptors are involved in autophagy regulation. While it is possible that other receptors are also involved in ammonia sensing, especially other Dopamine receptors, DRD3 is the first receptor discovered so far that senses ammonia and regulates autophagy.

We hope our work will be useful in alerting other researchers that they should pay extra attention to the freshness of their culture medium. The Glutamine-containing medium in Fig 1A was incubated at 37 degrees in the absence of cells. After our initial surprise at this phenomenon, we found that the supplier Sigma had tested Glutamine in medium in the absence of cells and found that it starts to be degraded at 37 degrees within one day, and the degradation increases over time (https://www.sigmaaldrich.com/content/dam/sigma-aldrich/docs/Sigma/Product_Information_Sheet/t075.pdf.). In contrast, GlutaMAX is much more stable and our work suggests that switching to GlutaMAX will be beneficial to other autophagy researchers.

Our study resolves some literature debate. Our experiments that compared different time points explained the lack of MTOR inhibition in Eng et al and Cheong et al’s studies where they used 24 hours incubation [5,11] while Harders et al observed moderate changes on MTOR pathway when they treated cells with ammonia for 4 hours [12]. In addition, the differential autophagic effects of ammonia at different concentrations were also consistent with the observation by Polletta et al [38]. Polletta et al’s study revealed that SIRT5 is an upstream component in ammonia generation in cells while our study focused on the downstream components of ammonia. It is likely that low concentrations of ammonia are sensed by DRD3, and possibly other membrane receptors (such as other dopamine receptors), which we are currently investigating, while high concentrations of ammonia diffuse through the membrane and change lysosome pH to block autophagic flux.

It should be noted that inhibition of MTOR activity is not the only reason for ammonia’s effect on DRD3 degradation because the MTOR inhibitor Rapamycin does not result in the same relocalization of DRD3 or dramatic increase of free GFP fragment. In fact, ammonia not only inhibits MTOR activity, but also changes MTOR localization, and this localization change is abolished after DRD3 RNAi. The relocalization of DRD3 onto the autophagosomes and the generation of free GFP fragment upon ammonia addition are mainly due to increased DRD3 internalization and subsequent degradation, in which PIK3C3/VPS34 plays important roles. In contrast, Rapamycin-induced autophagy is through direct inhibition of MTOR and it does not trigger DRD3 internalization from the cell surface. Therefore, ammonia and Rapamycin do not have identical phenotypes.

Millimolar concentrations of ammonia are frequently seen in acute hyperammonemia patients. We used lower millimolar amounts of ammonia, which is comparable to the blood ammonia concentrations in most clinical hyperammonemia patients (0.5–5 mM) as well as in tumor microenvironments (2–5 mM). Further investigations on ammonia’s action in other clinically relevant settings, such as neurons, will be critical to provide mechanisms about clinical consequences such as encephalopathy. It will be also helpful for studies of tumor microenvironment and autophagy-induced chemoresistance.

## Supporting Information

S1 Fig Ammonia solution gives a similar result as ammonium chloride. (DOCX)

S2 Fig Location of the GFP tag and the presence of a Flag tag do not affect GFP-DRD3’s response to ammonia. (DOCX)

S3 Fig General lysosomal/autophagic inhibitors induce much less GFP fragment from GFP-DRD3-Flag than NH_4_Cl does and GFP-DRD3-Flag do not respond significantly to rapamycin treatment. (DOCX)

S4 Fig NH_4_Cl does not directly inhibit MTOR activity *in vitro* but inhibit MTOR activity in cells. (DOCX)
