# Supplementary material for: Ammonia Induces Autophagy through Dopamine Receptor D3 and MTOR
Source: PLoS One. 2016 Apr 14;11(4):e0153526. doi: 10.1371/journal.pone.0153526 (PMC4831814; doi:10.1371/journal.pone.0153526)
Supplement: S1 Fig — (DOCX) [file pone.0153526.s001.docx]

**

**

**S1 Fig. Ammonia solution gives a similar result as ammonium chloride.** (A) Similar to ammonia chloride, ammonia solution also induces GFP fragments from HeLa-GFP-DRD3-Flag. HeLa-GFP-DRD3-Flag were treated with ammonium chloride or ammonia solution for 24 hours. (B) Ammonia solution induces obvious GFP fragments from HeLa-GFP-DRD3-Flag, but not HeLa-GFP-LC3-Flag. HeLa cells stably expressing GFP-DRD3-Flag or GFP-LC3-Flag cells were treated with ammonia solution for 24 hours. Experiments were repeated three times and representative Western blots are shown. Densitometric analysis was performed and quantification results were labeled below the corresponding blots.
