# Supplementary material for: Ammonia Induces Autophagy through Dopamine Receptor D3 and MTOR
Source: PLoS One. 2016 Apr 14;11(4):e0153526. doi: 10.1371/journal.pone.0153526 (PMC4831814; doi:10.1371/journal.pone.0153526)
Supplement: S2 Fig — (DOCX) [file pone.0153526.s002.docx]

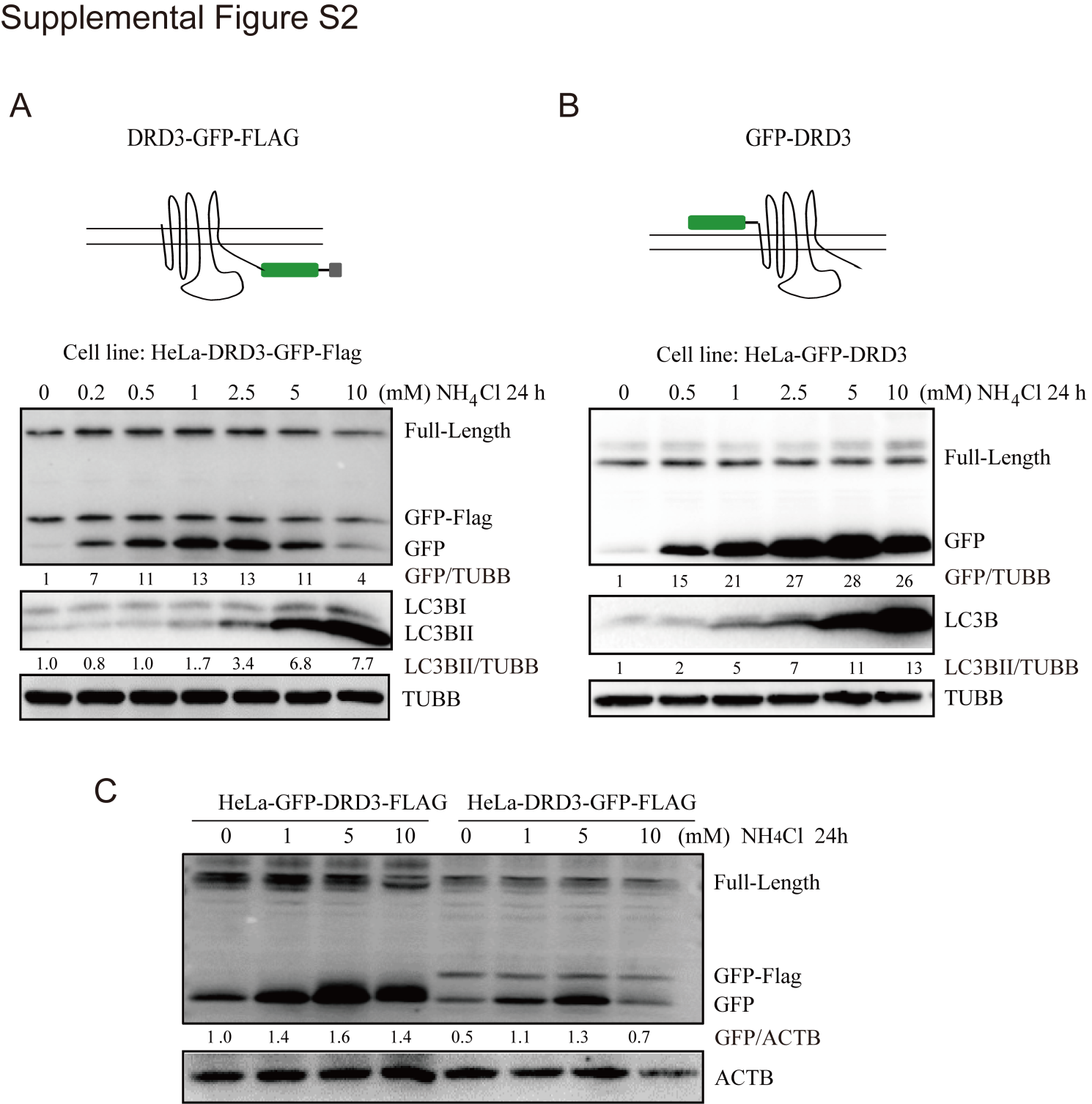


**S2 Fig. Location of the GFP tag and the presence of a Flag tag do not affect GFP-DRD3’s response to ammonia.** (A) HeLa-DRD3-GFP-Flag, (B) HeLa-GFP-DRD3, (C) HeLa-DRD3-GFP-Flag and HeLa-GFP-DRD3-Flag stable cell lines were treated with ammonium chloride. Experiments were repeated three times and representative Western blots with anti-GFP, or Actin antibodies are shown. Densitometric analysis was performed and quantification results were labeled below the corresponding blots.
