# Supplementary material for: Ammonia Induces Autophagy through Dopamine Receptor D3 and MTOR
Source: PLoS One. 2016 Apr 14;11(4):e0153526. doi: 10.1371/journal.pone.0153526 (PMC4831814; doi:10.1371/journal.pone.0153526)
Supplement: S3 Fig — (DOCX) [file pone.0153526.s003.docx]

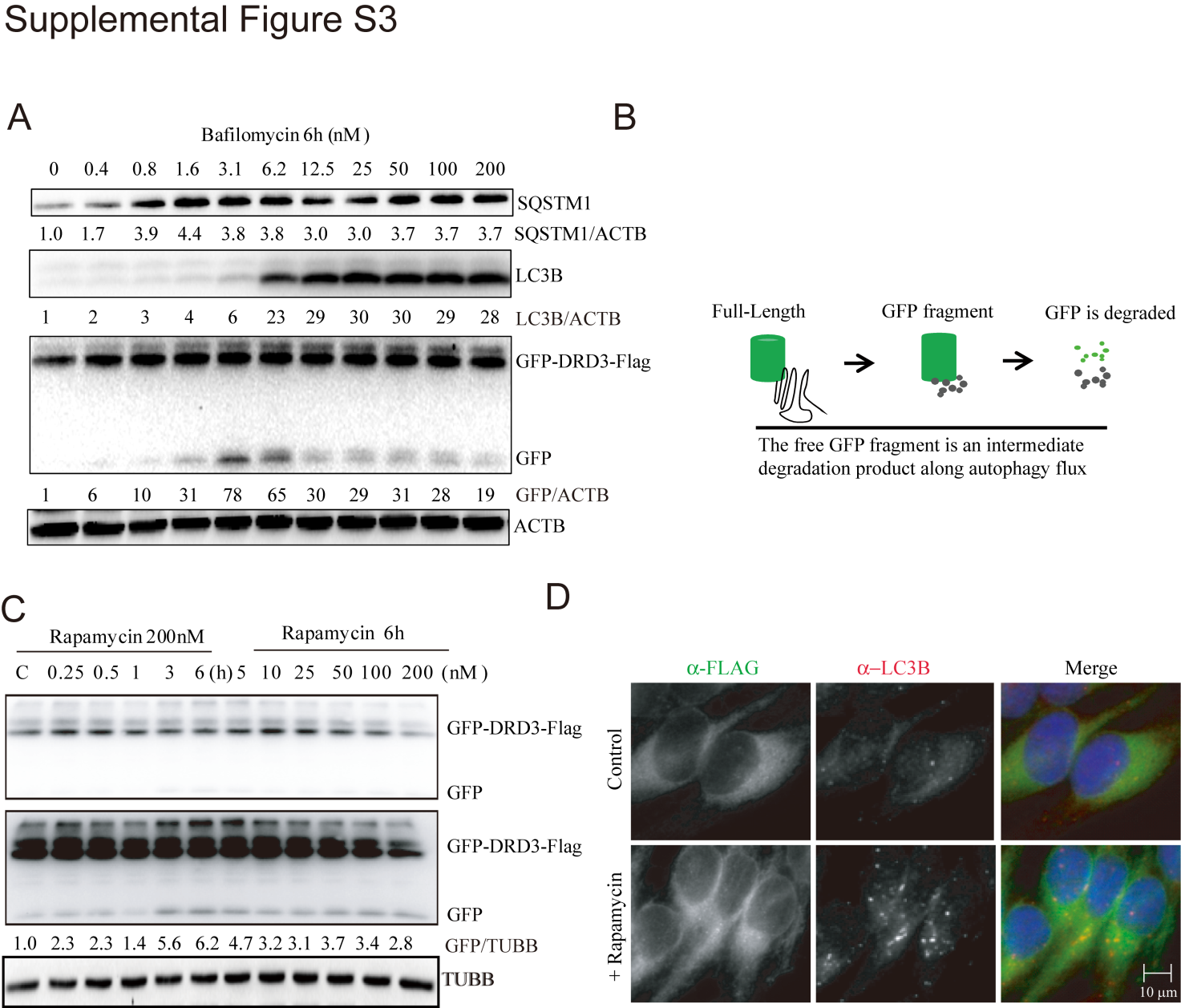


**S3 Fig. General lysosomal/autophagic inhibitors induce much less GFP fragment from GFP-DRD3-Flag than NH_4_Cl does and GFP-DRD3-Flag do not respond significantly to rapamycin treatment.** (A) Bafilomycin A1 induces much less GFP fragment from GFP-DRD3-Flag than NH_4_Cl does. HeLa cells stably expressing GFP-DRD3-Flag were treated with different concentrations of Bafilomycin A1 for 24 hours. Cells were harvested and lysed for Western blots. Experiments were repeated two times and representative Western blots with anti-GFP, SQSTM1, LC3B or Actin antibodies are shown. (B) Schematic illustration of the process of GFP fragment generation. (C) HeLa cells stable expressing GFP-DRD3-FLAG were cultured in different concentrations of rapamycin for indicated time. Representative Western blots with anti-GFP antibody (two different exposures) are shown. (D) Immunofluorescence of HeLa-GFP-DRD3-Flag cells treated with 200 nM rapamycin or DMSO, fixed in -20^o^C methanol and stained with anti-Flag and anti-LC3B antibodies. Representative Western blots are shown. Densitometric analysis was performed and quantification results were labeled below the corresponding blots.
