# Supplementary material for: Ammonia Induces Autophagy through Dopamine Receptor D3 and MTOR
Source: PLoS One. 2016 Apr 14;11(4):e0153526. doi: 10.1371/journal.pone.0153526 (PMC4831814; doi:10.1371/journal.pone.0153526)
Supplement: S4 Fig — (DOCX) [file pone.0153526.s004.docx]

**S4 Fig. NH_4_Cl does not directly inhibit MTOR activity *in vitro* but inhibit MTOR activity in cells.** (A) NH_4_Cl inhibits pS6K in HeLa and HCT116 cells. HeLa cells and HCT116 cells were incubated with increasing concentrations of NH_4_Cl for 1 hour. Representative western blots are shown. (B) *In vitro* kinase MTOR assay in the presence of different concentrations of NH_4_Cl or the positive control compound Torin 2, an MTOR inhibitor. Representative Western blots are shown. Densitometric analysis was performed and quantification results were labeled below the corresponding blots.
